# New quantitative trait loci for carotid atherosclerosis identified in an intercross derived from apolipoprotein E-deficient mouse strains

**DOI:** 10.1152/physiolgenomics.00099.2012

**Published:** 2013-03-05

**Authors:** Jessica S. Rowlan, Zhimin Zhang, Qian Wang, Yan Fang, Weibin Shi

**Affiliations:** Departments of Radiology & Medical Imaging and Biochemistry & Molecular Genetics, University of Virginia, Charlottesville, Virginia

**Keywords:** carotid atherosclerosis, dyslipidemia, mice, quantitative trait locus, stroke

## Abstract

Carotid atherosclerosis is the primary cause of ischemic stroke. To identify genetic factors contributing to carotid atherosclerosis, we performed quantitative trait locus (QTL) analysis using female mice derived from an intercross between C57BL/6J (B6) and BALB/cJ (BALB) apolipoprotein E (*Apoe*^−/−^) mice. We started 266 F_2_ mice on a Western diet at 6 wk of age and fed them the diet for 12 wk. Atherosclerotic lesions in the left carotid bifurcation and plasma lipid levels were measured. We genotyped 130 microsatellite markers across the entire genome. Three significant QTLs, *Cath1* on chromosome (Chr) 12, *Cath2* on Chr5, and *Cath3* on Chr13, and four suggestive QTLs on Chr6, Chr9, Chr17, and Chr18 were identified for carotid lesions. The Chr6 locus replicated a suggestive QTL and was named *Cath4*. Six QTLs for HDL, three QTLs for non-HDL cholesterol, and three QTLs for triglyceride were found. Of these, a significant QTL for non-HDL on Chr1 at 60.3 cM, named *Nhdl13*, and a suggestive QTL for HDL on ChrX were new. A significant locus for HDL (*Hdlq5*) was overlapping with a suggestive locus for carotid lesions on Chr9. A significant correlation between carotid lesion sizes and HDL cholesterol levels was observed in the F_2_ population (*R* = −0.153, *P* = 0.0133). Thus, we have identified several new QTLs for carotid atherosclerosis and the locus on Chr9 may exert effect through interactions with HDL.

stroke is the leading cause of disability in adults and the fourth most common cause of death in the United States (33). Ischemic stroke, resulting from the obstruction of blood flow to the brain, accounts for >80% of all stroke cases (4). Most of the ischemic strokes are caused by atherosclerosis in the carotid arteries. Carotid plaques directly or indirectly, through formation of thrombi, result in stenosis of the vessels and block the blood flow to the brain (24). Due to the ready accessibility and the close association with the brain, the carotid arteries are the most extensively studied vessels in vivo with ultrasonography. Patients with noticeable carotid stenosis have severely impaired cerebral blood flow and markedly increased risk for ipsilateral stroke (24, 26). For those with no obvious carotid stenosis, perspective studies also show a close association between the intima-media thickening and the risk of stroke (21, 25, 48). Intima-media thickening of the carotid arteries is an established parameter of subclinical atherosclerosis (9, 40).

Genetic studies of twin pairs and families have demonstrated the heritability of carotid atherosclerosis or common and internal carotid artery intima media thickness (34, 44, 60). However, identification of susceptibility genes involved has not been achieved. To date, robust and replicable associations in nonisolated populations are only limited to variants in the APOE gene (29). A recent meta-analysis of genome-wide association studies (GWAS) from the CHARGE consortium has identified a few common variants associated with carotid intima media thickness and plaque, including LRIG1, EDNRA, SLC17A4, PIK3CG, PINX1, ZHX2, APOC1, and LDLR (2). However, it is challenging to establish causality between the variants and the disease in humans due to small gene effect, complex genetic structure, and environmental influences.

A complementary approach to the identification of human disease genes is to use model organisms. One commonly used mouse model of atherosclerosis is the apolipoprotein E-deficient (*Apoe*^−/−^) mouse, which develops all phases of atherosclerotic lesions throughout the aorta and its branches (27). We have demonstrated a dramatic influence of genetic backgrounds on the development of carotid atherosclerosis in *Apoe*^−/−^ mouse strains (19). In an intercross derived from C57BL/6 (B6) and C3H/HeJ (C3H) *Apoe*^−/−^ mice, we performed quantitative trait locus (QTL) analysis and identified the first significant locus for carotid atherosclerosis (19). Like C3H.*Apoe*^−/−^ mice, BALB/cJ (BALB) *Apoe*^−/−^ mice are highly resistant to atherosclerosis (46). In this study, we generated a F2 population from B6.*Apoe*^−/−^ and BALB.*Apoe*^−/−^ mice to search for new loci contributing to carotid atherosclerosis and associated lipid traits.

## METHODS

### 

#### Mice.

B6.*Apoe*^−/−^ mice were purchased from the Jackson Laboratories, and BALB.*Apoe*^−/−^ mice at the N10 generation were generated in our laboratory. The creation of a female F_2_ population from the two *Apoe*^−/−^ mouse strains was as reported (59). Mice were started on a Western diet containing 21% fat, 34.1% sucrose, 0.15% cholesterol, and 19.5% casein (Harlan Laboratories, TD 88137) at 6 wk of age and maintained on the diet for 12 wk. Mice were bled once before initiation of the Western diet and once at the end of the feeding period. Blood samples were collected from overnight fasted mice through a retro-orbital vein puncture with the animals under isoflurane anesthesia. All procedures were performed in accordance with current National Institutes of Health guidelines and approved by the University Animal Care and Use Committees.

#### Plasma lipid analysis.

The measurements of total cholesterol, HDL cholesterol, and triglyceride were performed as reported previously (46). Non-HDL cholesterol was calculated as the difference between total and HDL cholesterol.

#### Quantitation of carotid atherosclerosis.

The distal portion of the left common carotid artery and its adjacent branches were harvested en bloc, embedded in OCT compound (Tissue-Tek), and processed, as we previously reported (19). Sections were stained with oil red O and hematoxylin and counterstained with fast green. Lesion areas were quantified using an ocular with a square-micrometer grid on a light microscope. The lesion areas of five sections with the largest readings were averaged for each mouse, and this average was used for statistical analysis.

#### Genotyping.

A total of 130 microsatellite markers covering all 19 autosomes and the X chromosome at an average interval of ∼12 cM were typed. Parental and F_1_ DNA served as controls for each marker.

#### Statistical analysis.

QTL analysis was performed using J/qtl software, as we previously reported (41, 42, 57). One thousand permutations of trait values were run to define the genome-wide logarithm of odds (LOD) score thresholds for significant and suggestive linkage to each trait. Loci that exceeded the 95th percentile of the permutation distribution were defined as significant (*P* < 0.05), and those exceeding the 37th percentile were suggestive (*P* < 0.63). Pair-wise genome scans were performed to find interacting loci affecting carotid lesions.

#### Prioritization of candidate genes.

The Sanger single nucleotide polymorphism (SNP) database (http://www.sanger.ac.uk/cgi-bin/modelorgs/mousegenomes/snps.pl) was searched to prioritize candidate genes for carotid lesion QTL that had been mapped in intercrosses derived from different parental strains. Probable candidate genes should possess one or more SNPs in coding or upstream promoter regions that are shared by the parental strains carrying the “high” allele but are different from the parental strains carrying the “low” allele.

## RESULTS

### 

#### Trait value frequency distributions.

**Fig. 1.**
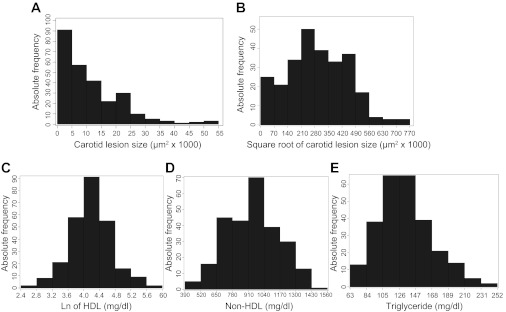
Frequency distributions of carotid atherosclerotic lesion sizes and plasma lipid levels in 266 female F_2_ mice fed a Western diet for 12 wk. The F_2_ mice were generated from an intercross between B6.*Apoe*^−/−^ and BALB.*Apoe*^−/−^ mice. *A*: carotid atherosclerotic lesion sizes; *B*: square root-transformed carotid atherosclerotic lesion sizes; *C*: LN (natural log)-transformed plasma HDL cholesterol levels; *D*: plasma non-HDL cholesterol levels; *E*: plasma triglyceride levels.

Values of atherosclerotic lesion sizes in the left carotid bifurcation of 266 F_2_ mice were distributed in the Pareto manner: the proportion of F_2_ mice with a lesion size ≤5,000 μm^2^/section is the highest and then decreases as lesion sizes increase (Fig. 1). After being square root-transformed, these values approach a normal distribution. Values of natural logarithm-transformed HDL cholesterol, non-HDL cholesterol, and triglyceride concentrations are approximately normally distributed. These data were then analyzed using J/qtl software to detect significant and suggestive QTLs affecting the traits.

#### Carotid atherosclerotic lesions.

**Fig. 2.**
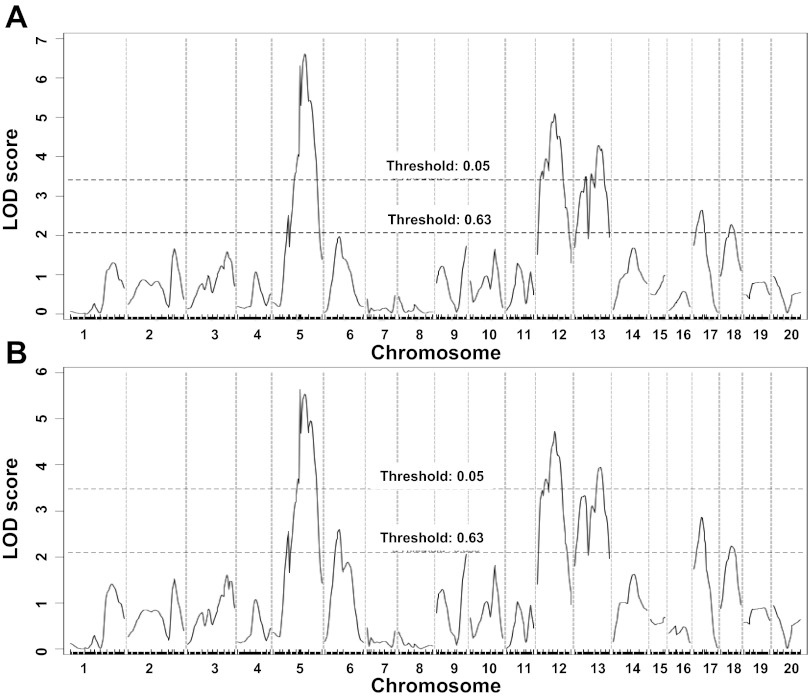
Genome-wide quantitative trait locus (QTL) analysis to search for loci influencing carotid lesion sizes in the F_2_ population derived from B6.*Apoe*^−/−^ and BALB.*Apoe*^−/−^ mouse strains. Chromosomes 1–20 are represented numerically on the *x*-axis. The relative width of the space allotted for a chromosome reflects the number of microsatellite markers typed for that chromosome. The *y*-axis represents the logarithm of the odds (LOD) score. Two horizontal dashed lines denote genome-wide thresholds for suggestive (*P* = 0.63) and significant (*P* = 0.05) linkage. The QTL analysis of original carotid lesion data with the nonparametric mode (*A*); the QTL analysis of square root-transformed carotid lesions using the parametric mode (*B*).

**Fig. 3.**
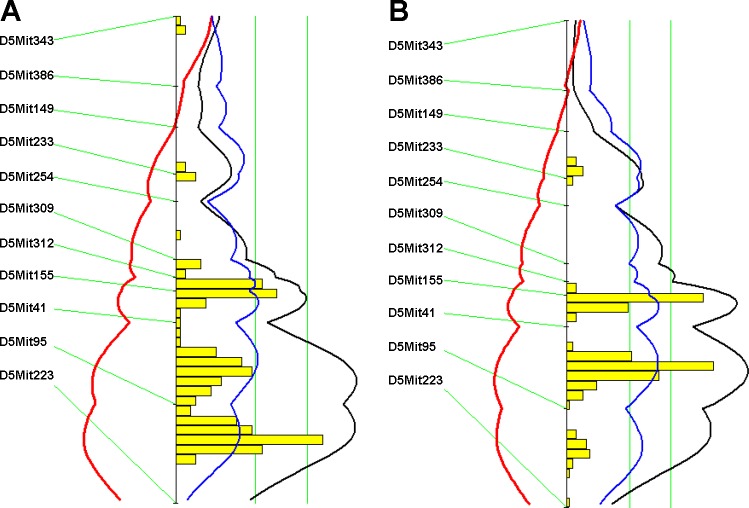
LOD score plots for carotid atherosclerotic lesions in the F_2_ population on chromosome 5. The plots were created with the interval mapping function of Map Manager QTX including a bootstrap test shown as a histogram estimating the confidence interval of a QTL. Two straight vertical lines on the plot represent the genome-wide significance thresholds for suggestive and significant linkage. The black line denotes the likelihood-ratio statistic calculated at 1-cM intervals. The blue line represents the effect of the B6 allele, and the red line represents the effect of the BALB allele. The interval mapping graph for the original carotid lesion data (*A*); the interval mapping graph for the squire root-transformed carotid lesion data (*B*). The histogram in both plots suggests the existence of 2 significant QTLs.

Genome-wide QTL analysis of either nontransformed carotid lesion sizes with the nonparametric mode or square root-transformed carotid lesion sizes with the parametric mode revealed three significant QTLs, located on chromosomes (Chr) 5, 12, and 13, and two suggestive QTLs, on Chr17 and Chr18, for carotid lesion sizes (Fig. 2). The QTL analysis of square root-transformed carotid lesions with the parametric mode also revealed two additional suggestive QTLs, located on Chr6 and Chr9. Details of the QTLs detected, including locus name, LOD score, 95% confidence interval (CI), peak location, genome-wide significance *P* value, high allele, and mode of inheritance are presented in Table 1. The significant QTL on Chr12 and the suggestive QTL on Chr6 replicated two previously reported QTLs (19). The other QTLs were novel. The Chr5 locus peaked at 56.7 cM and had a significant LOD score of 6.6 and a genome-wide significant *P* value of 0.0001. We named it *Cath2* to represent a locus for mouse carotid atherosclerosis. Interval mapping plots for Chr5 using either nontransformed carotid lesion data or square root-transformed lesion data showed two distinct peaks with each surpassing the significant LOD score threshold (Fig. 3), indicating the presence of two loci for the trait on the chromosome. The bootstrap test, a statistical method for defining the CI of a QTL using simulation (49), also indicated the existence of two QTLs in the region for the trait. However, because the two loci overlapped in the CI and both exhibited a dominant effect from the B6 allele, we designated them as a single QTL. The Chr13 locus had a significant LOD score of 5.1 and a genome-wide significance *P* value of 0.001. Its peak appeared at 49 cM. We named it *Cath3*. The Chr6 locus had a suggestive LOD score of 2.6 and peaked at 35.5 cM. It replicated a suggestive QTL for carotid lesions mapped in the B6.*Apoe*^−/−^ × C3H.*Apoe*^−/−^ intercross (19). We named it *Cath4*. For all three significant QTLs and one suggestive QTL on Chr17, the B6 allele was responsible for increased lesion sizes and the BALB allele accounted for reduced lesion sizes. In contrast, for the Chr18 locus the BALB allele was associated with increased lesion sizes and the B6 allele was associated with decreased lesion sizes (Table 2). The two suggestive QTLs on Chr6 and Chr9 exhibited an underdominance manner of inheritance in that F_2_s with the heterozygous genotype had a significantly smaller or larger lesion size than those homozygous for the B6 or BALB allele.

**Table 1.** Significant and suggestive QTLs for carotid atherosclerosis and plasma lipid levels in F_2_ mice derived from B6 Apoe^−/−^ and BALB.Apoe^−/−^ mice

Locus Name	Chr	Trait	LOD	Peak, cM	95% CI	*P* Value	High Allele	Mode of Inheritance
*Cath2*	5	carotid lesion (nonparametric)	**6.615**	56.68	48.46–66.68	0.0001	B6	dominant
*Cath1*	12	carotid lesion (nonparametric)	**5.095**	32.59	16.59–44.59	0.001	B6	additive
*Cath3*	13	carotid lesion (nonparametric)	**4.292**	48.99	18.99–59.69	0.009	B6	additive
	17	carotid lesion (nonparametric)	2.641	22.14	8.14–28.14	0.248	B6	recessive
	18	carotid lesion (nonparametric)	2.274	30	16–40	0.455	BALB	additive
*Cath2*	5	carotid lesion (parametric)	**5.64**	48.68	48.46–70.68	0.001	B6	dominant
*Cath4*	6	carotid lesion (parametric)	2.595	35.5	25.5–57.5	0.295		underdominance
	9	carotid lesion (parametric)	2.064	69.87	17.8–69.87	0.652		underdominance
*Cath1*	12	carotid lesion (parametric)	**4.734**	32.59	14.59–44.59	0.001	B6	additive
*Cath3*	13	carotid lesion (parametric)	**3.95**	50.99	14.99–60.99	0.019	B6	additive
	17	carotid lesion (parametric)	2.861	22.14	8.14–28.14	0.175	B6	recessive
	18	carotid lesion (parametric)	2.234	30	18–42	0.508	BALB	additive
*Hdlq5*	1	HDL	**4.291**	82.31	74.31–92.31	0.008	BALB	additive
*Hdlq17*	9	HDL	**5.917**	23.8	19.8–27.8	0.0001	BALB	additive
*Lipq2*	13	HDL	2.428	59.69	44.99–67.21	0.415	BALB	dominant
*Hdlq29*	17	HDL	2.924	29.73	23.97–40.14	0.166	BALB	dominant
*Hdlq32*	19	HDL	2.509	40.3	18.3–46.3	0.351	BALB	recessive
	20	HDL	2.763	51.49	35.49–76.75	0.217		underdominance
*Nhdlq13*	1	non-HDL	**5.93**	60.31	54.31–82.31	0.0001	BALB	dominant
*Cq1*	1	non-HDL	**5.85**	74.3	69.3–82.3	0.0001	BALB	additive
*Pnhdlc1*	6	non-HDL	2.617	53.5	33.5–67.5	0.281	B6	additive
*Tglq1*	1	triglyceride	**6.069**	74.31	68.31–89.0	0.0001	BALB	additive
*Tgq4*	7	triglyceride	2.28	41.37	33.37–65.37	0.458	B6	additive
*Tgl1*	8	triglyceride	2.481	69.7	31.7–72.18	0.347		underdominance

The newly identified quantitative trait loci (QTLs) were named if they were significant or if they replicated previously reported suggestive ones. The nomenclature of *Cath* was for carotid atherosclerosis QTLs. The newly identified QTLs were underlined to easily distinguish them from known ones. Logarithm of odds (LOD) scores were obtained from genome-wide QTL analysis using J/qtl software. The significant LOD scores are highlighted in boldface. The suggestive and significant LOD score thresholds were determined by 1,000 permutation tests for each trait. Suggestive and significant LOD scores were 2.064 and 3.410, respectively, for carotid atherosclerosis (nonparametric); 2.096 and 3.475 for square root-transformed carotid atherosclerosis (parametric); 2.090 and 3.600 for HDL cholesterol, 2.081 and 3.513 for non-HDL cholesterol, and 2.066 and 3.679 for triglyceride. Confidence interval (CI) of 95% in cM defined by a whole genome QTL scan. The *P* values reported represent the level of genome-wide significance as they were generated based on genome-wide permutation tests. Mode of inheritance was defined according to allelic effect at the nearest marker of a QTL.

**Table 2.** Effects of B6 and BALB alleles in different QTLs on carotid atherosclerosis and plasma lipids in the intercross between B6.Apoe^−/−^ and BALB.Apoe^−/−^ mouse strains

Locus Name	Chr	Trait	Peak Marker	Peaks, cM	BB	CC	BC	*P* Value
*Cath2*	5	carotid lesion (nonparametric)	D5Mit41	56.68	116832.9 ± 109325.59 (*n* = 68)	71120.7 ± 92371.5 (*n* = 55)	129914.7 ± 104129.0 (*n* = 131)	2.05E-03
*Cath1*	12	carotid lesion (nonparametric)	D12Mit201	32.59	146489.2 ± 110520.6 (*n* = 52)	72933.6 ± 95294.4 (*n* = 59)	115870.9 ± 100169.5 (*n* = 154)	6.66E-04
*Cath3*	13	carotid lesion (nonparametric)	D13Mit147	48.99	146242.1 ± 104769.9 (*n* = 58)	79884.7 ± 93992.1 (*n* = 77)	116396.0 ± 103879.1 (*n* = 130)	8.20E-04
	17	carotid lesion (nonparametric)	D17Mit66	22.14	149831.4 ± 114851.4 (*n* = 65)	102302.1 ± 107115.5 (*n* = 76)	99280.7 ± 90916.3 (*n* = 115)	3.92E-03
	18	carotid lesion (nonparametric)	D18Mit35	30	79168.0 ± 75581.7 (*n* = 65)	133860.3 ± 118030.6 (*n* = 63)	120239.4 ± 105365.8 (*n* = 134)	6.06E-03
*Cath2*	5	carotid lesion (parametric)	D5Mit155	48.68	114473 ± 108135 (*n* = 72)	64025 ± 90979 (*n* = 51)	129348 ± 101158 (*n* = 137)	5.38E-04
*Cath4*	6	carotid lesion (parametric)	D6Mit243	35.5	114825 ± 101947 (*n* = 64)	145489 ± 127531(*n* = 80)	89273 ± 78934 (*n* = 121)	7.18E-04
	9	carotid lesion (parametric)	D9Mit279	69.87	81594 ± 76681 (*n* = 62)	109173 ± 99109 (*n* = 59)	128345 ± 112776 (*n* = 142)	1.14E-02
*Cath1*	12	carotid lesion (parametric)	D12Mit201	32.59	146489 ± 110521 (*n* = 52)	72934 ± 95294 (*n* = 59)	115871 ± 100170 (*n* = 154)	6.66E-04
*Cath3*	13	carotid lesion (parametric)	D13Mit147	50.99	146242 ± 104770 (*n* = 58)	79885 ± 93992 (*n* = 77)	116396 ± 103879 (*n* = 130)	8.20E-04
	17	carotid lesion (parametric)	D17Mit66	22.14	149831 ± 114851 (*n* = 65)	102302 ± 107116 (*n* = 76)	99281 ± 90916 (*n* = 115)	3.92E-03
	18	carotid lesion (parametric)	D18Mit35	30	79168 ± 75582 (*n* = 65)	133860 ± 118031 (*n* = 63)	120239 ± 105366 (*n* = 134)	6.06E-03
*Hdlq5*	1	HDL	D1Mit354	82.31	65.3 ± 34.1 (*n* = 57)	99.2 ± 31.6 (*n* = 68)	71.4 ± 12.7 (*n* = 128)	2.47E-05
*Hdlq17*	9	HDL	D9Mit285	23.8	61.1 ± 37.9 (*n* = 71)	100.5 ± 55.5 (*n* = 52)	74.8 ± 43.6 (*n* = 135)	3.76E-06
*Lipq2*	13	HDL	D13Mit148	59.69	63.5 ± 41.5 (*n* = 56)	84.9 ± 52.2 (*n* = 82)	77.9 ± 43.6 (*n* = 123)	2.82E-02
*Hdlq29*	17	HDL	D17Mit20	29.73	61.8 ± 35.8 (*n* = 69)	84.5 ± 51.9 (*n* = 66)	80.8 ± 46.2 (*n* = 121)	6.12E-03
*Hdlq32*	19	HDL	D19Mit90	40.3	67.6 ± 36.5 (*n* = 64)	93.7 ± 60.3 (*n* = 68)	72.2 ± 39.9 (*n* = 128)	1.52E-03
	20	HDL	DXMit84	51.49	68.1 ± 35.9 (*n* = 79)	68.7 ± 54.5 (*n* = 43)	84.5 ± 48.3 (*n* = 138)	1.92E-02
*Nhdlq13*	1	non-HDL	D1Mit495	60.31	845.3 ± 195.0 (*n* = 65)	994.1 ± 196.5 (*n* = 65)	978.6 ± 215.1 (*n* = 123)	2.35E-05
*Cq1*	1	non-HDL	D1Mit354	80.32	855.7 ± 199.6 (*n* = 57)	1026.2 ± 215.8 (*n* = 68)	945.2 ± 206.0 (*n* = 128)	4.20E-05
*Pnhdlc1*	6	non-HDL	D6Mit102	53.5	1010.9 ± 197.1 (*n* = 66)	895.2 ± 210.9 (*n* = 73)	953.3 ± 217.5 (*n* = 118)	5.87E-03
*Tglq1*	1	triglyceride	D1Mit354	74.31	117.2 ± 31.7 (*n* = 57)	145.1 ± 38.0 (*n* = 68)	135.2 ± 31.8 (*n* = 128)	2.82E-05
*Tgq4*	7	triglyceride	D7NDS1	41.37	143.8 ± 33.2 (*n* = 55)	124.2 ± 29.3 (*n* = 64)	134.2 ± 36.5 (*n* = 134)	7.29E-03
*Tgl1*	8	triglyceride	D8Mit13	69.7	128.3 ± 33.1 (*n* = 65)	123.3 ± 29.7 (*n* = 64)	140.1 ± 35.7 (*n* = 127)	2.62E-03

Measurements are expressed as means ± SD. The units for these measurements are: μm^2^/section for carotid atherosclerotic lesions and mg/dl for plasma lipid levels. BB, homozygous for B6 alleles at the linked peak marker; CC, homozygous for BALB alleles; BC, heterozygous for B6 and BALB alleles at the peak marker. The number in the parentheses represents the number of progeny with a specific genotype at a peak marker. ANOVA was used to determine the significance level (*P* value) of differences for a specific phenotype among progeny with 3 different genotypes at a specific marker.

**Fig. 4.**
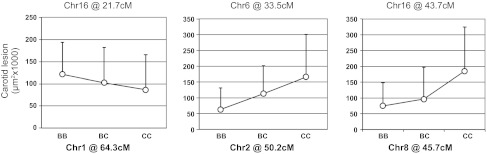
Interacting QTLs detected by pairwise genome scans. BB, homozygous for B6 alleles; CC, homozygous for BALB alleles; BC heterozygous. The *y-*axis denotes carotid lesion sizes. Error bars represent SD.

Pairwise genome scans were performed to search for interacting loci affecting carotid lesion sizes (Fig. 4). Significant interactions were detected between the Chr1 locus at 64 cM and the Chr16 locus at 21.7 cM, between the Chr2 locus at 50.2 cM and the Chr6 locus at 33.5 cM, and between the Chr8 locus at 45.7 cM and the Chr16 locus at 43.7 cM. There were no main-effect QTLs for carotid atherosclerosis at any of these loci alone. However, in those F_2_ mice that were homozygous for the B6 allele at 21.7 cM on Chr16, the Chr1 locus exhibited an additive effect from the B6 allele in increasing lesion sizes. In F_2_ mice homozygous for the B6 allele at 33.5 cM on Chr6, the BALB allele of Chr2 locus at 50.2 cM increased lesion sizes in an additive manner. The BALB allele of Chr8 locus at 45.7 cM also increased lesion sizes when the Chr16 locus was homozygous for the BALBc allele at 43.7 cM.

#### Plasma lipid levels.

**Fig. 5.**
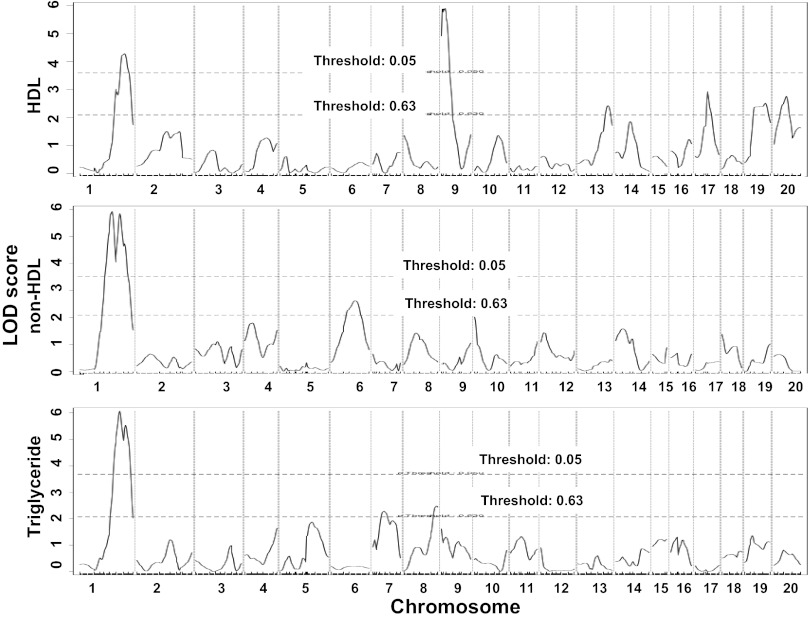
Genome-wide QTL analysis to search for loci affecting plasma levels of HDL cholesterol (*top*), non-HDL cholesterol (*middle*), and triglyceride (*bottom*) in the F_2_ population. Chromosomes 1–X are represented numerically on the *x*-axis, and the *y*-axis represents the LOD score.

**Fig. 6.**
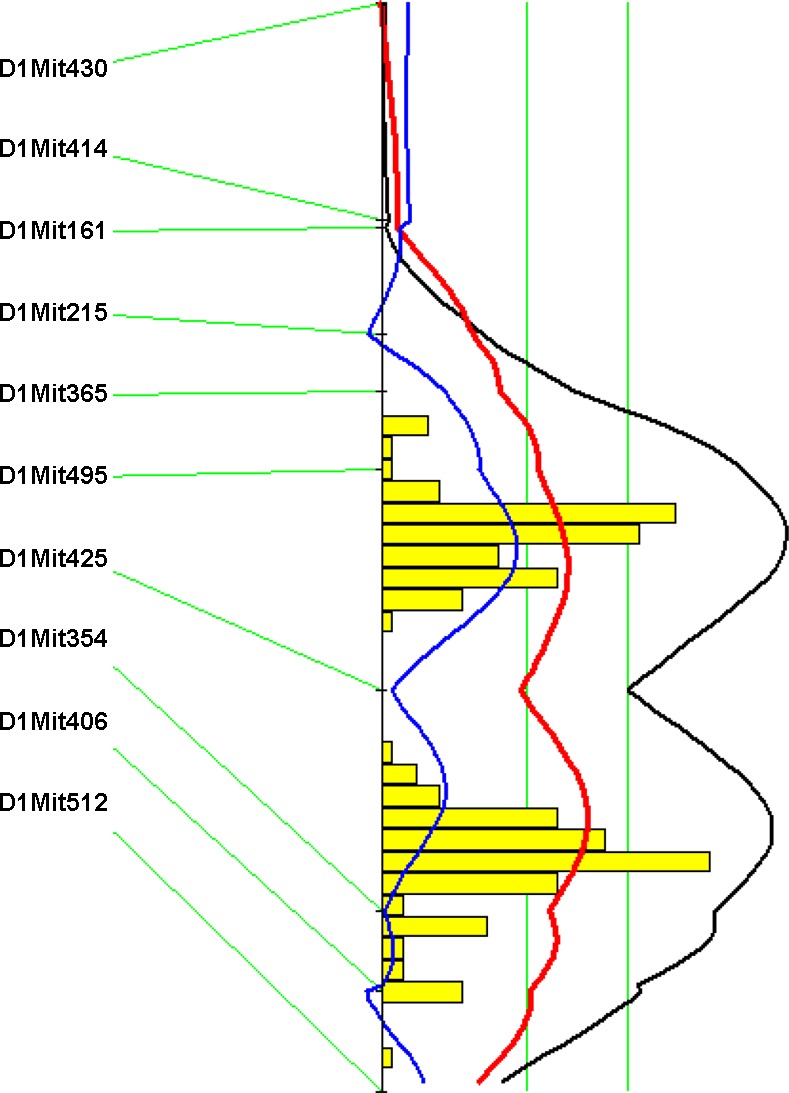
LOD score plot for non-HDL cholesterol levels on chromosome 1. The plot was created with the interval mapping function of Map Manager QTX, including a bootstrap test shown as a histogram estimating the confidence interval for the QTL. The plot indicates the existence of 2 significant QTLs for the trait on chromosome 1.

Genome-wide QTL analysis showed that plasma HDL cholesterol, non-HDL cholesterol, and triglyceride levels were each controlled by multiple loci (Fig. 5, Table 1). For HDL, two significant QTLs, located on Chr1 and Chr9, and four suggestive QTLs on Chr13, Chr17, Chr19, and the X chromosome were identified. The two significant QTLs on Chr1 and Chr9 replicated *Hdlq5* and *Hdlq17,* respectively, which had been mapped in several crosses (53). The Chr9 locus was partially overlapping with the suggestive locus for carotid atherosclerosis (Table 1). The suggestive locus on Chr13 partially overlapped with *Lipq2*, a locus identified in B6.C-H25C × BALB/cJ F_2_ mice (55). The suggestive QTLs on Chr17 and Chr19 were overlapping in the CI with *Hdlq29* and *Hdlq32,* respectively, identified in a NZB × SM F_2_ cross (16). The locus on the X chromosome was novel. For non-HDL cholesterol, two significant QTLs on Chr1 and 1 suggestive QTL on Chr6 were identified. LOD score plot for Chr1 showed two distinct peaks, located ∼14 cM apart (Fig. 6). The distal QTL peaked at 74.3 CM, overlapping with *Cq1*, a locus originally identified in a B6 × KK-Ay intercross for plasma cholesterol concentrations (43). The proximal peak occurred at 60.3 cM with a significant LOD score of 5.93. We named *Nhdlq13* to represent a significant QTL for non-HDL cholesterol in the mouse. The suggestive QTL on Chr6 replicated *Pnhdlc1* mapped in a B6 × CASA/Rk intercross (37). Plasma triglyceride levels were controlled by one significant QTL on Chr1 and two suggestive QTLs on Chr7 and Chr8. The Chr1 and Chr7 QTLs replicated *Tglq1* and *Tgq4*, respectively, loci originally mapped in a NZB × SM F_2_ cross (30). The Chr8 QTL overlapped with *Tql1* mapped in a KK/Ta × (BALB/cxKK/Ta) backcross (39).

#### Relationships of plasma lipids with carotid atherosclerosis.

**Fig. 7.**
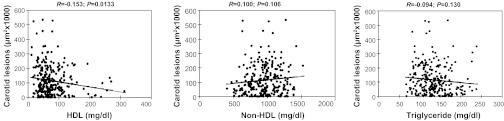
Scatterplots showing the relationship of carotid lesion sizes with lipid levels in the F_2_ population. Each point represents an individual value of an F_2_ mouse. The correlation coefficient (*R*) and significance (*P*) are shown. Plasma levels of HDL cholesterol were significantly correlated with the sizes of carotid lesions but plasma levels of non-HDL cholesterol and triglyceride were not.

Associations of carotid lesion sizes with plasma lipid levels were analyzed using the F_2_ population. A small but statistically significant inverse correlation was found between plasma HDL cholesterol levels and carotid lesion sizes in the F_2_ mice (*R* = −0.153, *P* = 0.0133; Fig. 7). Mice with higher HDL cholesterol levels tended to develop smaller carotid lesions. No statistically significant correlation was observed between non-HDL cholesterol levels and carotid lesion sizes, although there was a trend toward statistical significance (*R* = 0.100, *P* = 0.106). There was also a trend toward a significant inverse correlation between plasma triglyceride levels and carotid lesion sizes (*R* = −0.094, *P* = 0.130).

#### Probable candidate genes for Cath1.

As *Cath1* has also been mapped in an intercross between B6.Apoe^−/−^ and C3H.Apoe^−/−^ mice (19), we conducted a haplotype analysis using the Sanger SNP database to prioritize candidate genes for the QTL (Table 3). A few candidate genes underneath the linkage peak of *Cath1* were found, including *Egln3* (55.3 Mb), *Eapp* (55.7 Mb), *Mipol1* (58.3 Mb), *Foxa1* (58.6 Mb), *Clec14a* (59.3 Mb), and *Mis18bp1* (66.2 Mb). These candidates contain one or more nonsynonymous polymorphisms that are shared by the low allele strains (C3H and BALB) but are different from the high allele strain (B6) at the locus. *Egln3*, encoding EGL nine homolog 3, possesses the prolyl hydroxylase activity, which is required to suppress the activation of NF-κB (8). *Mipol1*, *Foxa1*, and *Mis18bp1* are three genes associated with cell growth and tumor development. Recent GWAS have identified several new loci for coronary heart disease that are also associated with cancer (11, 36). *Clec14a* encodes an endothelial cell-specific protein that plays a role in cell-cell adhesion. Knockdown of *Clec14a* with siRNA suppressed cell migratory activity in vitro (32).

**Table 3.** Haplotype analysis to prioritize candidate genes for Cath1

			High Allele	Low Allele	
Gene	Chr	Position	C57BL/6	BALB	C3H
*Egln3*	12	55304356	**A**	**G**	**G**
	12	55304602	T	G	G
	12	55304677	G	A	A
*Eapp*	12	55771990	T	C	C
	12	55772852	G	a	a
	12	55773051	A	t	t
	12	55774107	A	T	T
	12	55774193	T	C	C
	12	55774467	C	T	T
	12	55796707	G	**A**	**A**
*Gm16382*	12	55802602	T	**g**	**G**
*2700097O09Rik*	12	56146803	T	C	C
	12	56149876	G	**C**	**C**
	12	56181074	C	a	A
*Prps1l3*	12	58331404	A	G	G
*Mipol1*	12	58331419	C	T	T
	12	58404379	G	A	A
	12	58426585	A	**G**	**G**
	12	58426610	A	**G**	**G**
	12	58558185	C	T	T
	12	58567779	T	G	G
	12	58597592	C	T	T
*Foxa1*	12	58642920	C	c/g	c/g
	12	58643254	T	**C**	**C**
*Ttc6*	12	58677129	G	**C**	**C**
*Clec14a*	12	59365857	T	C	C
	12	59366183	T	C	C
	12	59366468	G	A	A
	12	59368777	T	**C**	**C**
	12	59369326	T	**C**	**C**
	12	59369975	C	G	G
	12	59369979	G	C	C
*Ctage5*	12	60232475	T	C	C
*Fscb*	12	65572716	G	**A**	**A**
	12	65573078	G	**C**	**C**
	12	65573952	C	**a**	**A**
	12	65574300	G	**A**	**A**
*Klhl28*	12	66043857	C	G	G
	12	66043917	G	C	C
	12	66043927	A	G	G
	12	66044072	T	G	G
	12	66044105	A	G	G
	12	66051133	C	**T**	**T**
*Fancm*	12	66214956	A	**T^+^**	**T^+^**
	12	66231329	A	**C^+^**	**C^+^**
	12	66231384	A	**T^+^**	**T^+^**
	12	66231423	A	**C^+^**	**C^+^**
*Mis18bp1*	12	66249896	A	**a/c**	**a/c**
	12	66249994	C	**T**	**T**
	12	66253824	T	C	C
	12	66253867	C	A	A
	12	66253871	T	A	A
	12	66273454	T	A	A
	12	66273538	A	G	G

Analysis was performed using Sanger SNP database (http://www.sanger.ac.uk/cgi-bin/modelorgs/mousegenomes/snps.pl). Boldface denotes nonsynonymous nucleotide substitution and blue denotes synonymous nucleotide substitution.

## DISCUSSION

In this study, we used an F_2_ population derived from an intercross between two phenotypically divergent *Apoe*^−/−^ mouse strains to localize chromosomal regions contributing to carotid atherosclerosis and associated lipid traits. We have identified three significant QTLs and four suggestive QTLs for carotid atherosclerosis and 10 QTLs for plasma lipid levels from the cross. Moreover, we have observed significant associations of carotid lesion sizes with plasma HDL cholesterol levels in the F_2_ population.

*Cath1* was the first QTL for carotid atherosclerosis identified in an intercross between B6.*Apoe*^−/−^ and C3H.*Apoe*^−/−^ mice (19). It was located on mouse chromosome 12 at 25 cM with the B6 allele contributing to an increased lesion size in female mice. The present study replicated this QTL and also a suggestive locus on chromosome 6, now named *Cath4*, mapped in the previous cross. The CI of *Cath1* corresponds to human chromosome 14q12–14q24, a region that has shown linkage to carotid intimal medial thickness and premature coronary artery disease (6, 51). As *Cath1*.*Pik3cg* (32.8 Mb) is a gene in the region that has been found to be associated with carotid intima media thickness and plaque in humans on GWAS (2). There are multiple polymorphisms between B6 and BALB mice within and upstream of the *Pik3cg* gene. We also conducted a haplotype analysis using parental strains in intercrosses that led to detection of *Cath1*, and found several probable candidate genes underneath the linkage peak of *Cath1*, including *Egln3* (55.3 Mb), *Mipol1* (58.3 Mb), *Foxa1* (58.6 Mb), *Clec14a* (59.3 Mb), and *Mis18bp1* (66.2 Mb).

A new major QTL for carotid atherosclerosis mapped in this cross was *Cath2*, which had a highly significant LOD score. This locus was coincident with a fasting plasma glucose QTL, *Bglu13,* mapped in this cross (59). A significant correlation between fasting plasma glucose and carotid lesion sizes was also observed in the cross (data not shown), suggesting a possibility that both phenotypes were regulated by the same underlying QTL gene(s). *Hnf1a* (114.9 Mb), encoding hepatocyte nuclear factor 1α, is the most promising candidate in this regard. It is located underneath the linkage peaks of *Cath2* and *Bglu13*. One A/G SNP in exon 9 between B6 and BALB leads to amino acid substitution (P580R) in the *Hnf1a* protein. In humans, *Hnf1a* mutations are the most common cause of maturity-onset diabetes of the young (38). Polymorphisms in the *Hnf1a* gene are associated with risk for Type 2 diabetes and coronary heart disease (31, 50). The mouse chromosome 5 region from 48 to 67 cM corresponds to chromosomal regions of 4q22 and 12q24 in humans. The 12q24 region is associated with coronary heart disease (5, 36), carotid intima media thickening (6), metabolic syndrome (12, 58), and Type 1 and Type 2 diabetes (47, 50, 56). Other probable candidate genes for the QTL include *Adamts3* (90.1Mb), *Ankrd17* (90.6 Mb), and *Cxcl5* (91.1 Mb). The *Adamt* proteases are secreted enzymes that regulate extracellular matrix turnover by degrading specific matrix components. Recent studies have suggested a role for the proteases in inflammation and atherosclerosis (35). *Ankrd17* encodes an ankyrin repeat protein that mediates protein-protein interactions. Ankyrin repeat proteins are involved in a variety of physiological processes, such as cell cycle control and inflammatory response (17, 54).

A new significant locus for carotid atherosclerosis, named *Cath3*, was found on chromosome 13 at 49 cM. This QTL was colocalized with an HDL locus, *Lipq2*, originally mapped in a B6.C-H25c × BALB/cJ cross (55) and replicated in the current cross. Moreover, a significant correlation was found between carotid lesion sizes and HDL cholesterol levels in the F_2_ population. *Bhmt2* (94.4 Mb), which is located underneath the linkage peak of *Cath3*, is a promising candidate gene. It encodes the betaine-homocysteine methyltransferase, an enzyme involved in regulating the susceptibility to acetaminophen-induced liver toxicity (20). This enzyme may also affect the susceptibility to high fat diet-induced liver damage. High-fat diet results in a spectrum of liver damages, ranging from simple steatosis, to active inflammation, to advanced fibrosis and cirrhosis (3). B6.*Apoe*^−/−^ mice develop severe hyperglycemia, dyslipidemia, and systemic inflammation in response to a high-fat diet, whereas BALB.*Apoe*^−/−^ mice are resistant to these conditions (18, 46). In humans, steatotic liver disease is associated with increased prevalence of cardiovascular disease (29). *F2rl1* (96.2 Mb) and *F2rl2* (96.4 Mb) are also promising candidate genes in the region. They are involved in many processes relevant to atherosclerosis, such as inflammatory response, endothelium dysfunction, diet-induced obesity, and insulin resistance (1, 15, 45).

We found that QTLs in distal chromosome 1 were responsible for the major variations in plasma HDL, non-HDL cholesterol, and triglyceride levels of the cross. This finding is consistent with our observation made in two separate crosses between B6.*Apoe*^−/−^ and C3H.*Apoe*^−/−^ mice (19, 42). *Apoa2* has been identified as a QTL gene on distal chromosome 1 contributing to variations in plasma lipid levels of mice (52). We recently have demonstrated that *Soat1* is also a QTL gene contributing to naturally occurring variations in plasma lipid levels of mice (22). In the current cross, we have observed two distinct peaks of the linkage curve for non-HDL cholesterol on chromosome 1 with the distal peak appearing at 74.3 cM and the proximal peak at 60.3 cM. The bootstrap test, a statistical method for defining the CI of QTLs using simulation (49), also indicated the existence of two QTLs for the trait on chromosome 1. We named the proximal QTL *Nhdlq13* to represent a new locus for non-HDL cholesterol in the mouse. *Lct* (130.2 Mb), encoding lactase, is the only candidate gene in the QTL interval that could explain variation in plasma lipid levels.

A significant QTL for HDL was mapped to chromosome 9 at 24 cM in the current cross. This QTL was overlapping with *Hdlq17* mapped in a B6 × 129S1/SvImJ intercross (13), *Chol10* mapped in a 129S1/SvImJ × RIIIS/J intercross (23), and *Phdlc1* mapped in B6 × CASA/RkJ intercross (37). There are several promising candidate genes in the region, including *St3gal4* (34.8 Mb), *Sorl1* (41.7 Mb), *Sc5d* (42 Mb), *Phldb1* (44.5 Mb), *Sik3* (46 Mb), *Apoa1* (46 Mb), *Fam55b* (48.1 Mb), and *Fam55d* (48.1 Mb).

Although dyslipidemia is a well-recognized risk factor for ischemic stroke, the present study of F_2_ mice has only revealed a weak correlation between carotid atherosclerotic lesion sizes and plasma HDL cholesterol levels. Unexpectedly, no correlations were observed between carotid lesions and non-HDL cholesterol or between carotid lesions and triglyceride levels. A previous study of F_2_ mice derived from B6.*Apoe*^−/−^ and C3H.*Apoe*^−/−^ mice also demonstrated no significant associations between carotid lesion sizes and plasma lipoprotein levels (19).

In summary, we have identified multiple QTLs contributing carotid atherosclerosis and plasma lipid levels in hyperlipidemic *Apoe*^−/−^ mice. Most of the QTLs identified for carotid atherosclerosis in this study are novel. The coincidence of QTLs for carotid atherosclerosis and HDL as well as the correlation between the traits suggests that they may be controlled by the same gene in the QTL interval.

## GRANTS

This work was supported by National Heart, Lung, and Blood Institute Grant HL-82881.

## DISCLOSURES

No conflicts of interest, financial or otherwise, are declared by the author(s).

## AUTHOR CONTRIBUTIONS

Author contributions: J.S.R., Z.Z., Q.W., Y.F., and W.S. performed experiments; J.S.R., Z.Z., Q.W., Y.F., and W.S. analyzed data; J.S.R., Z.Z., Q.W., Y.F., and W.S. approved final version of manuscript; W.S. conception and design of research; W.S. interpreted results of experiments; W.S. prepared figures; W.S. drafted manuscript; W.S. edited and revised manuscript.
